# Acceptability of cool roofs: a qualitative study in Nouna, Burkina Faso

**DOI:** 10.1186/s12889-025-23806-w

**Published:** 2025-08-27

**Authors:** Kate Bärnighausen, Moubassira Kagone, Alina Herrmann, Guillaume Compoaré, Adama Gansane, Siaka Debe, Maquins Odhiambo Sewe, Jose Guillermo Cedeno Laurent, Sandra Barteit, Martina Anna Maggioni, Raissa Sorgho, Till Bärnighausen, Ali Sié, Aditi Bunker

**Affiliations:** 1 School of Public Health, The University of the Witwatersrand, Johannesburg, South Africa; 2 Heidelberg Institute of Global Health (HIGH), Faculty of Medicine and University Hospital, Heidelberg University, INF 130.3, Heidelberg, 69120 Germany; 3 Centre de Recherche en Santé de Nouna (CRSN), Ministry of Health, Nouna, Burkina Faso; 4 Department of Public Health and Clinical Medicine, Umeå University, Umeå, Sweden; 5 Environmental Health and Occupational Health Sciences Institute, School of Public Health, Rutgers University, Rutgers, USA; 6 Institute of Physiology, Center for Space Medicine and Extreme Environment Berlin, Charité - Universitätsmedizin Berlin, Berlin, Germany; 7 Department of Biomedical Sciences for Health, University degli Studi di Milano, Milan, Italy

**Keywords:** Cool roof, Acceptability, Preferences, Adaptation, Qualitative, Climate change

## Abstract

**Background:**

Structural passive cooling interventions such as cool roofs are used to reduce indoor ambient temperature. However, it is unknown how acceptable and desirable cool roof technology is in rural low-income settings in sub-Saharan Africa, where home occupants are exposed to rising indoor temperatures.

**Methods:**

We engaged 48 participants in four focus group discussions to explore the factors influencing the acceptability of “cool roofs” in Nouna, Burkina Faso. We analysed the data using reflexive thematic analysis. We structured our findings using the acceptability framework developed by Sekhon, Cartwright and Francis (2017), which comprises seven components: affective attitude, burden, perceived effectiveness, ethicality, intervention coherence, opportunity costs, and self-efficacy.

**Results:**

Our participants described an environment of extreme heat and the need for adaptation strategies to reduce the temperature within their homes. The cool roofs would be deemed acceptable if they were affordable, effective in reducing heat, and aligned with values around self-efficacy, particularly in relation to local production and ownership.

**Conclusion:**

Providing communities with technical information regarding how the cool roof functions and can be maintained may support uptake via acceptability. Desirability of the roof may be achieved via a combination of highlighting the indoor cooling of the roof as reported by users, sharing of results with the community so that they have an insight into the effects of the roof, and feedback regarding the products useability and durability.

## Introduction

Global warming has led to a rise in surface temperatures increasing human exposure to outdoor and indoor heat in sub-Saharan Africa [1]. The resulting heat stress is one of the leading causes of heat-related morbidity and mortality in sub-Saharan Africa [2]. Heat stress is associated with adverse pregnancy outcomes, and non-communicable diseases such as cognitive decline, cardiovascular disease, cancer, chronic respiratory disease and diabetes [3, 4]. The resulting morbidity and mortality negatively impacts economic and social productivity, further impoverishing already climate vulnerable communities [5]. Cooling strategies that are sustainable, inexpensive and that require minimal resources may support resource-constrained communities to cope with increasing heat exposures and extreme heat events [6, 7]. Sustainable cooling strategies may be especially important in sub Saharan Africa, where rural communities have limited capacity to adapt to harmful temperature exposures that are projected to increase.

In Burkina Faso, ranked 131 st out of 135– by GDP– of the poorest countries in the world [8], surface temperatures could increase by between 1.9 and 4.2 °C by 2080 compared to pre-industrial levels [9]. This is largely attributable to global warming trends, but is also associated with local deforestation of woodlands for cattle herding, biomass for fuel and farming [10]. While these predictions of surface temperature rises are alarming, more significant is the exposure of home occupants to variability of indoor heat levels which– depending on the structural type of the house– can be 10 °C higher than the outdoor temperature [9]. In the majority of households in rural Burkina Faso, physiological heat stress indicators measured as hourly heat index values to determine the human-perceived equivalent temperature - by a combination of air temperature and humidity - revealed that harmful thresholds of 40–54 °C are frequently exceeded, and this threshold is almost permanently exceeded during all seasons except winter [9].

Most rural houses are constructed with low-cost materials such as local mud– known as ‘banco’ or ‘beaten earth’ and corrugated metal sheets. While in some countries metal sheets have been shown to reduce both mosquito survival [11] and indoor heat levels during the night, in Burkina Faso, these metal sheets have limited cooling capacity and warm the indoor– often poorly ventilated - space, exposing inhabitants to indoor temperatures that are the cause of the afore mentioned acute and long-term health issues [12, 13]. In rural Burkina Faso, many of the banco roofs traditionally used have been replaced with these corrugated metal sheets, owing to the high maintenance of banco and inadequate protection against rain. People living in poor housing conditions in rural Burkina Faso are already marginalised as they lack the means to adopt expensive adaptation technologies which often require access to electricity, such as air conditioning, further exacerbating their susceptibility to heat stress [14].

Existing adaptive technologies in Africa– such as shading through vegetation [15] - would require an extensive reforesting scheme, as deforestation is a major issue in Burkina Faso [16]. Other technologies such as shading, double glazing, wind catchers and green roofs - designed to reduce indoor temperatures are commonplace and functional where adaptive capacity is greater than in rural Burkina Faso. Moreover, cooling strategies like these are highly dependent on the local climate. Wind catchers, which deliver fresh air to a house’s occupants, require relatively large open spaces in housing, such as terraces or large windows [17], are effective in sub-tropical areas where there’s a significant temperature drop from day to night, but ineffective in tropical or semi-arid regions– like Burkina Faso - with consistent temperatures throughout the day and the night. Double glazing of windows, which is a well-established method of reducing heat transfer between the in- and outside of a building, is not an option in rural Burkina Faso, because houses have either no or very small windows and the other insulating (walls and doors) or cooling (ventilation) elements of the house are insufficient for double glazing to function. Green roofs, which reduce indoor heat by absorbing sun rays, require frequent rain or constant irrigation [18], which is not possible in the dry season. Similarly, evaporative cooling using a fountain requires the availability of ample water, but may exacerbate problems with mosquitos, is also not possible in the dry season, are very expensive and challenging to construct and maintain [19].

However, passive cooling via cool roof technology is a promising option for adaptation in rural settings where other cooling methods are not possible. Cool roofs have two important properties — high solar reflectance and thermal emittance– that work synergistically to reduce indoor temperature. The high solar reflectance enables solar energy to be reflected by the roof, and high thermal emittance enables the roof surface to radiate absorbed solar energy. Cool roofs work on the basic premise that a roof which reflects direct sunlight and radiates heat can stay cooler than a roof that absorbs sunlight [20].

While studies examining cool roof technologies have taken place in contexts with different climates and roof types, they provide an insight into the potential of this adaptation strategy. In Jamaica, the application of cool roof coating on a 36 m^2^ uninsulated concrete roof resulted in a 4 °C reduction of internal air temperature in April compared to before the coating was applied [21]. In Gujarat, India, cool roof coating of a 9.3 m^2^ galvanized roof surface with a white coating (solar reflectance index (SRI) 0.9) resulted in a 3.1 °C temperature reduction in comparison to a white roof of the same size and orientation [22]. Cool roofs installed on flat roofed residential homes in Andalucia, Spain were found to reduce overall electricity consumption by 2% and save €59 million annually, whilst preventing emissions of 136,000 tons of carbon dioxide (CO_2_) from electricity generation [22].

While these findings are compelling, the acceptability of any adaptation technology is fundamental to its effectiveness, and can often be unrelated to the actual effect of the intervention [23]. Little is known about how best to introduce the cool roof in a way that is acceptable considering preferences such as which colour and surface material to offer [24], what information people need and whether people feel the cool roof is a viable solution to address the heat in their homes. Should the intervention be effective, understanding these factors– among others– will help refine and support the application of cool roofs in Burkina Faso and other climate resource-constrained settings globally.

We are conducting a randomized controlled trial (RCT) in the Nouna Health and Demographic Surveillance System (HDSS) in partnership with the Centre de Recherche en Santé de Nouna (CRSN). The RCT, in Nouna, Burkina Faso, examines the effectiveness of cool roof technology on health, environmental and economic outcomes in 600 homes. Our formative qualitative study engaged four communities across the Nouna HDSS to understand the acceptability and preferences of cool roof technology prior to trial implementation. We present qualitative findings from Focus Group Discussions (FGDs) designed to elicit the acceptability of cool roofs, the best methods for the application of the technology and the technical preferences of cool roofs for homes.

## Methods

### The cool roof intervention

The cool roof RCT sample was drawn from 124,256 individuals living in 14,305 homes in 59 villages. We randomly selected 1200 individuals living in 600 homes in 25 villages. One female and one male participant from 600 households were enrolled in the study (*n* = 1200 participants). Of the 600 homes, we randomly assigned 300 homes to receive a cool roof and 300 homes to receive no modification [25]. Considering we had two prominent roof types with different heat-absorbing properties, we randomly selected 12 homes in each village with mud (banco) roofs and 12 with metal sheet roofs. The distribution of these 600 households across the control and intervention arms were evenly allocated between mud brick and sheet metal roof houses [25].

The primary study outcome is heart rate and we have collected three measurements monthly from every participant [25]. We have also collected data to assess the effects of cool roofs on (i) secondary environmental outcomes, which includes ambient temperature and humidity (measured using iButton sensors), and (ii) secondary health outcomes such as blood pressure, body temperature, heat-related outcomes, blood glucose, sleep, cognition and mental health. To understand the effect of the roof on biological indicators associated with heat stress, we also randomly selected 152 participants [intervention, *n* = 76 (38 women, 38 men) versus control, *n* = 76 (38 women, 38 men)] to receive a smartwatch that continuously monitors heart rate, sleep, and activity [25]. Heat exposure leads to vasodilation, which increases blood flow to the periphery to enable radiative cooling– placing strain on the heart to pump harder and faster to maintain blood circulation (increase cardiovascular demand and reduce heart filling pressure). Furthermore there is loss of fluid from sweating which also compromises cardiovascular function [26]. The qualitative component presented in this manuscript consists of formative research to understand community perceptions of the cool roofs and how they can be improved. The cool roof intervention was informed by research relating to indoor heat stress within the community and draws on participatory research principles to ensure the outcomes of the intervention are in line with health needs of communities in rural Burkina Faso. There is currently no cool roof manufacturing in Burkina Faso, so materials for our trial were shipped from three manufacturing companies– Sika AG, SOPREMA SAS and Engineered Polymer Solutions B.V.– that are members of the European Cool Roof Council (ECRC)– a non-governmental organization promoting cool roof uptake across Europe. Technical experts from the three companies trained local painters from Nouna on their application methods, who we subsequently employed for implementing cool roofs in our RCT. Details on the cool roof intervention can be accessed in our study protocol [25].

### Study setting

This study was conducted in the Nouna HDSS which is managed by the CRSN, within the Kossi province in North-Western Burkina Faso where the cool roof RCT is taking place. This region has the semi-urban district capital of Nouna and many surrounding rural villages which have populations of hundreds to only several inhabitants. The climate in the north of Burkina Faso is semi-arid with annual mean temperatures of up to 29 °C and high rates of evapotranspiration [27]. In this northerly region of the country, the annual precipitation sum is around 500 mm, the majority of which falls during the rainy season between June and September [28]. The broader Sudano-Sahelian regional climate features a wet bulb temperature in excess of 40 °C for an average of 200 days per year for approximately 4–6 h a day [28].

### Design

A formative qualitative study using focus group discussions (FGDs) designed to inform implementation of the cool roof RCT.

### Sample

We purposively selected *n* = 48 participants to participate in 4 FGDs (2 with women, 2 with men participants) for heterogeneity in age (> 18 years), ethnic group, experience living in houses with mudbrick (banco) or sheet metal roofing. Our sample size was calculated to achieve sufficient data power [29] and thematic saturation [30].

### Data collection

From February 20th 2020 to March 04th 2020 we conducted four FGDs. FGD moderators were experienced qualitative researchers from Burkina Faso educated to undergraduate degree level. Our study team designed the data collection tools, including an FGD cover sheet to capture participant details, an informed consent sheet, a participant information sheet and a FGD guide. The FGD guide was drafted by the study team with input from the local team at the CRSN. The FGD guide (Appendix 1) was designed to elicit broad and open discussion regarding participants’ perceptions of cool roof technology use and application in rural Burkina Faso. We specifically asked questions regarding preferences of alternative cool roof approaches, including different household members’ preferences (i.e., men and women), as well as cool roof work processes and design features. We used both visual and artefactual props in the FGDs including photographs of the predominant types of local homes with cool roofs and provided the participants with samples of variants of the cool roof materials to gain their immediate reactions (Appendix 1). We asked questions regarding the preferences of cool roof coatings including the colour, texture and smell. Before FGDs took place, we obtained written informed consent from all participants. We conducted FGDs in French, Dioula and or Bwamou, which were audio and video recorded and lasted between 90 and 120 min. We conducted debriefings after each interview to refine lines of enquiry, to enable a space for reflexive discussion and to triangulate key findings [31].

### Data analysis

**Fig. 1:**
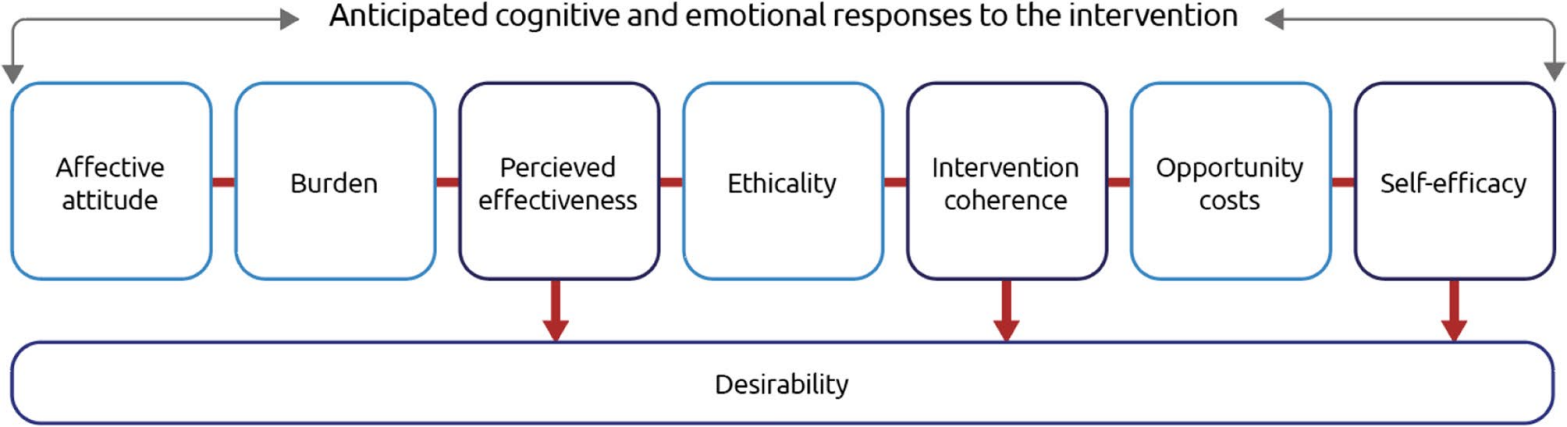
The adapted acceptability framework as defined by Sekhon, Cartwright and Francis [33]

All FGDs were transcribed in English and checked for accuracy by the FGD moderators. Transcripts, interviewer notes and debriefings were managed using NVivo Pro 12 and analysed using the tenets of Reflexive Thematic Analysis (RTA) [32]. This approach is appropriate for our study because RTA is theoretically flexible, and is considered useful for understanding peoples experiences, views and perceptions [32]. Our RTA process was recursive and followed six stages of analysis. Two data analysts independently read and re-read transcripts for data familiarisation, then inductively analysed the transcripts, and together reviewed where codes were similar or diverged. This process was followed until coding was complete. We presented the main codes to the research team and discussed where reoccurring codes could build our core themes. After this meeting, we identified our inductive codes as mostly building themes relating to acceptability. The primary data analyst then revised the codebook and independently developed a distinct thematic scheme which followed the tenets of the acceptability construct by Sekhon, Cartwright and Francis (2017)– a peer reviewed acceptability framework for interventions [23, 33]. Here, acceptability is defined as a multi-faceted construct, represented by seven component constructs: affective attitude, burden, perceived effectiveness, ethicality, intervention coherence, opportunity costs, and self-efficacy. We allocated our inductive codes to these seven component constructs, presented to the study team, refined and finalised for interpretation and writing up (Fig. 1). This thematic scheme was triangulated with the debriefing notes and observational notes taken during data collection [34].

## Results

We present an overview of individual characteristics (gender, age, occupation) and household characteristics representing 48 participants that were part of FGDs (Table 1). We present our themes within the Sekhon, Cartwright and Francis (2017) [23, 33] acceptability framework (Fig. 1).

**Table 1:** Socio-demographic characteristics of the study population

Characteristic	Number (%)
Women	19 (47.5)
Men	21 (52.5)
Age group	
30–40 Years	14 (35)
41–50 Years	12 (30)
51–60 Years	7 (17.5)
61–70 Years	5 (12.5)
71+ Years	2 (5)
Occupation	
Barber	1 (2.5)
Butcher	1 (2.5)
Dress maker	1 (2.5)
Farmer	20 (50)
Mason	2 (5)
Retired	3 (7.5)
Sweeper	1 (2.5)
Trader	11 (27.5)
Roof type	
Banco	2 (5)
Sheet metal	28 (70)
Banco and metal	10 (25)
Total	40 (100)

Participants described an environment of debilitating heat in Nouna and explain the impact extreme temperatures have on their ability to sleep, to conduct essential daily tasks and their health. When discussing the problems with heat, most participants said a major issue was the temperature within their homes, citing it as “a disease” (woman, farmer) and “suffering” (woman, trader). To best mitigate this problem, participants said they would shift their activities– when cool enough– that would normally take place inside the house to outside. Although this was in some ways effective at reducing the burden of certain types of heat (closeness as in, the heat is encroaching upon them, having to “wander around at night, just looking for some air” (woman, trader)), the outside ambient temperature was also described as “burning” (woman, farmer), and therefore related to distress. An unintended consequence of moving activities outside– including sleep– is that participants are exposed to mosquitos, which are vectors for disease. Participants associated the heat exposure with the loss of tree coverage in the immediate area and the changing climate with the lack of “mutual respect, solidarity and humanism” (man, farmer) displayed by humans. Although sheet metal roofing was described as socially desirable, in comparison to banco roofs it was consistently portrayed as being the source or exacerbating heat within the household. Having a technology that could reduce the temperature within their homes was seen, therefore, as essential.

*“If you notice*,* all of us who were forty-six years old that year*,* we all experienced some hardship. In the old days when we were children*,* our houses were made of clay with planks*,* our mothers lit pieces of tree trunks in our houses and no children got sick. But as soon as we grew up*,* me with my forty-six years old*,* we had houses made of tin*,* and we noticed that there are many illnesses nowadays. This is due to the use of sheet metal. They cause a lot of problems. It contains heat and cold too and you don’t know where to put your head.”* Man, Farmer.

*“We can also say that there is climate change*,* because other fools*,* we were more comfortable in our houses with the wooden roofs*,* clay roofs. Even in hot weather we didn’t have any problems*,* but now everything is made of sheet metal. Even in the countryside there are not even any more trees to make the clay houses*,* everything has become made of sheet metal and it gives off more heat than our clay houses. This is a change for us.”* Woman, Trader.

Following the acceptability construct as defined by Sekhon, Cartwright and Francis (2017) [23, 33], acceptability is defined as a multi-faceted construct, represented by seven component constructs: affective attitude, burden, perceived effectiveness, ethicality, intervention coherence, opportunity costs, and self-efficacy. As the participants had not yet received the intervention, our results reflect the anticipated cognitive and emotional responses to the intervention. We modified the framework by building an additional desirability component, which highlights where our findings within perceived effectiveness, intervention coherence and self-efficacy (in purple, Fig. 1) will likely support making the cool roof desirable. The desirability component is described in the discussion section.

### Affective attitude

Affective attitude is defined as ‘how an individual feels about the intervention’ [23, 33]. In most cases, participants described their feelings about cool roofs in relation to how others would perceive it and the wider communities’ perspective regarding the product. They frequently made reference to the switch from banco houses with mud roofs, to banco houses with sheet metal roofs, because this was how they could explain community momentum around something that was socially desirable. The attitude was, if one person had something that others did not, it would stimulate positive interest. Furthermore, hearing from others– such as neighbors - or seeing with your own eyes, that the cool roof technology actually worked, would make people trust and feel positively about the new product and want to be part of the intervention.

*It’s when you see something in another person that you become interested in it. If we start and we see that it’s a good thing*,* I can find out and come and tell M (anonymised) that it’s a good initiative*,* if there is a witness who confirms what I say*,* and so on*,* the news circulates and the population will adhere because the crowd has positively appreciated it. But until it’s experienced for people to see*,* we can’t believe in quality. We’ll have to experience it ourselves first*,* if it’s good*,* we’ll be able to circulate the good news.* Man, Farmer.

*In our opinion*,* if it is first applied on our roofs and it may be successful*,* other people will ask us questions about its origin and we will be able to tell you that you are in charge of it. We’ll tell them how to acquire it. When they too try it and it works*,* a lot of people will join. When those who make it know that it works well*,* they will venture to produce more and we pray to God that there will be no difficulty in their profession*,* that their work will prosper so that we can all easily acquire.* Woman, Trader.

When shown the cool roof product, participants explained how they felt about the intervention in relation to preferences regarding the roof. Participants first reactions to seeing the product included their preferences and feelings regarding how reflective the roof is, the texture and smell of the cool roof sample. Participants also inquired about sound and asked if the cool roof reduces the sound of rainfall. To a lesser extent, colour preferences were also discussed, and whether the colour would be visible on the sheet metal roof. Although some felt the colour was largely irrelevant, others felt that bringing a new, visible colour to the roof would be beneficial as it would show that there was something new bringing the reduction in temperature, and not the sheet metal alone.

*As everyone has a choice*,* if not in my opinion*,* the white colour is also the colour of the metal sheet*,* so you won’t even know there’s a change on the metal sheet but you have a favourable change of temperature in the house. If it’s to decorate*,* to embellish it*,* you can use another colour. For me*,* this is not necessary.* Man, Farmer.

*We have to change the colour so that people know that sheet metal alone does not change anything*,* we have applied it on the sheet metal and it gives freshness to the people*,* happiness to the people.* Man, Farmer.

### Burden

Burden is defined as the ‘perceived amount of effort that is required to participate in the intervention’ [23, 33]. In our data, participants talk about burden in relation to the initial cost of the product, the cost to maintain the cool roof and the courage to try something new. Although paying for something meant the product was likely to be good, it had to be a price that was considered appropriate for the product and within reach of those with limited financial means. There could be, as with the introduction of the sheet metal roofing, some social pressure to access the cool roof. This burden may be because the cool roof acts as an indication of financial means, signalling elevated social status. This kind of burden was also thought to be dynamic, as there would be some periods of time when funds could be accessed to buy or maintain the cool roof, and other times when they could not. Building the courage to participate was also described as a kind of burden, yet with the help of God and if the product was felt to be successful, this burden would be overcome.

*Nothing is free nowadays*,* but the price has to be well studied and it has to be within the reach of the population. For example*,* if you build your house for fifty thousand francs and you are told that you have to pay one hundred thousand francs to protect your roof. Will you be able to honour it?* Woman, Trader.

*We’ve seen it*,* apparently that’s good*,* but the cost*,* we have to be on the cost. If it’s not too expensive we can jump into it. We are afraid of expensive objects. We Africans believe that things of quality are expensive*,* but if it is extremely expensive*,* people of my calibre will not be able to pay.* Woman, Farmer.

*If they say it’s really beneficial*,* you pay your contribution according to your means to benefit from it*,* but if you don’t have the means*,* you won’t be able to force it. Here are the tin roofs that are gradually growing everywhere*,* that’s the means*,* the sister was saying that she doesn’t have the means*,* but there are tin houses in her concession*,* but not all the houses are made of tin. That’s what I’m talking about. There are days when she will be able to afford to put the tin on the rest*,* it is little by little.* Woman, Farmer.

*It all depends on the financial means. It’s like goods*,* you can take a good for the first time and you worry because you don’t know if it will work*,* but if you go out with it*,* with God’s help*,* a person can buy it and thinks it’s wonderful*,* they can spread the news and another one can come to you. That’s how it is*,* if you want to do something nowadays*,* you have to have the courage. That’s what it’s all about. So have the courage to do it*,* please God it’s going to work.* Woman, Farmer.

### Perceived effectiveness

Perceived effectiveness is defined as the ‘extent to which an individual feels the intervention will achieve its purpose’ [23, 33]. Participants spoke of their health in relation to the heat and explained that the excessive temperature is making them “sick” and “bringing disease”. They understood that the cool roof technology is supposed to be effective in relieving heat, thus improving their health and wellbeing. If the cool roof technology would prevent heat associated illness, it would be an effective intervention. However, one could observe both high hopes for the intervention as well as doubts about its effectiveness. Participants were wary of products that were seen as coming from outside and could be promoted by door-to-door campaigns. Products from “white people” (Man, Farmer) were not always in the best interests of the communities in Burkina, and participants feared that it could be something that may harm rather than improve their wellbeing.

*People like anything that makes them feel better. For example*,* if you are sick*,* you suffer even when you go to the health centre*,* you are sick*,* you can pay a health worker to come and take care of you at home and you are quiet in your house*,* less sick. The truth is that if it is something serious and could be implemented*,* everyone will adhere to it.* Man, Farmer.

*There is no problem*,* if it happens to be simple and does not create other difficulties in my house*,* there is no problem. But*,* if it will create other problems for me*,* I will not accept it*,* because you have done a bad job.* Man, Farmer.

*In my opinion*,* the population will adhere*,* because*,* whatever the price of paint*,* it can’t be worth the price of sheet metal. If you think about the heat*,* you don’t even often feel like going home at night because of the heat*,* and if they say they have found a solution against this heat*,* you will adhere.* Man, Farmer.

*I have already experienced a similar situation in my family*,* as you often give products to children in collaboration with health workers*,* door-to-door*,* I have my older brother who was one of those who used to work in the countryside*,* he broke into a concession in the Mossi district to give products to a child*,* the next morning the child fell ill and succumbed. His parents say that they will summon my brother and that he must go to prison because he came to poison their children with bad products. They were told that it was the law that allowed them to campaign and he was accompanied by the major of the health centre. It’s situations like that that make them afraid of things from white people.* Man, Farmer.

The main point of effectiveness was, that if the cool roof technology was cooling, it would be considered effective. Yet we found both doubts about the effectiveness of the cool roofs, as well as high hopes towards the technology, which might exceed its actual effects.

### Ethicality

Ethicality is defined as the extent to which the intervention is a ‘good fit with an individuals’ value system’ [23, 33]. We found the value system– within the context of the cool roof FGDs - alongside the need to modernize or adapt to the changing climate because the situation around heat was worsening. The value attached to traditional banco houses was clear via the preference and acceptance that this was a “comfortable” form of housing, but deforestation– leading to less protection from rain and the sun– meant that change was inevitable. The cool roof therefore, was ‘value’ appropriate for this moment in time, where rising temperatures and metal sheets, (although socially desirable), were heating homes in a way not felt before.

*What can I say*,* as it is modernization now*,* otherwise in the past our houses were made of beaten earth*,* during the heat the inside was cool during the cold*,* the climate was favourable*,* it was neither hot nor cold.* Woman, Trader.

*We should keep our black traditions*,* it’s very important*,* but it’s the fact that it’s flowing and the banco is of better quality*,* there’s no longer a qualified hand in this matter like the people of the past.* Woman, Farmer.

*But I know that in any case*,* in the past it was interesting*,* but nowadays it is said that there are no more trees*,* the banco houses were really comfortable. Everybody knows*,* we used to sleep there in peace*,* but nowadays*,* the metal sheets are getting hot.* Woman, Farmer.

*As soon as you are a little bit rich*,* you unhook your house to put metal sheets in it. On the other hand*,* if it’s a banco house*,* you’re at peace but people call you poor*. Woman, Trader.

Values relating to the health and happiness of children and family were clearly expressed by our participants, with the cool roof seeming to be a good fit with their value system of reducing the suffering of children and family members. Childrens’ suffering featured as a major component of the descriptions of heat stress, and therefore, their relief– as an expected result of the cool roof intervention - featured as the most acceptable ethical reason to participate.

*If I could have this on my roof*,* we would be at peace. We will not have to shower frequently because of the heat*,* the insomnia and the crying of the children because of the heat*,* everything will stop. Everyone will sleep in peace and wake up in peace.* Woman, Trader.

*It (the roof) is to prevent the heat from raging in our homes and also during the cold so that our children do not suffer.* Woman, Trader.

*But if it brings peace to my family*,* I will say it is a noble task. You can come and check it with your cameras when you come back*,* and I will also go out and tell my comrade that what you have done there as a job is impeccable.* Man, Farmer.

### Intervention coherence

Intervention coherence is the ‘extent to which the participant understands the intervention and how it works’ [23, 33]. Much of the described coherence of the cool roof intervention related to the quality of the product. Participants said they would accept the cool roof if it was of high quality and had questions regarding whether the quality of the product would wane over time. In a context where participants have hands on experience with building houses and installing two main types of roofs in their homes (banco ‘beaten earth’or sheet metal) or in neighbours’ homes, quality was gauged by the roofs ability to keep rainwater and dirt from entering the inside of the building. The metal roofs were seen to accomplish this and were also considered to be easier to maintain. Sheet metal was therefore the preference of most participants. However banco roofs were considered superior for keeping homes cool. If the cool roof achieved cooling on either roof, it would be considered effective. Quality was also associated with cost, and in this regard expectations of quality were tied with an understanding of the price of a sheet metal roof with the additional cost of a cool roof, and the level of quality this provided.

*Something of quality sells itself*,* when it is implemented and it has been installed on the roof of a dwelling and it has been successful*,* if people look at it*,* it is like the ceiling*,* a lot of people build nowadays they put ceiling*,* the one who can afford it*,* puts ceiling*,* the one who can’t afford it*,* he is satisfied with his roof made of sheet metal only.* Man, Farmer.

*The fibres that you are going to install before you paint the roof*,* won’t they deteriorate over time like the rainwater that falls on the roof?* Woman, Trader.

*It’s always clean and shiny. Whatever the colour*,* the most important thing is that the product is of high quality.* Woman, Farmer.

### Self-efficacy

Self-efficacy– within the acceptability construct - is defined as ‘the participant’s confidence that they can perform the behaviour(s) required to participate in the intervention’ [23, 33]. Self-efficacy was mostly expressed via where the cool roof was made, and that local production would allow participants to actively participate in the intervention. Participants explained that the cool roof would be more acceptable if produced locally because this would ensure local people would benefit from employment in the production and distribution of the technology. Local production would also build a sense of ownership and therefore, a sense of trust in the product, as this would allow the roofs to be seen as for the people in the area, and not just for the “white man” when he visited Africa. Participants explained that if those working in the production used the roofs themselves, it would be a good sign that this was a product that should be adopted by the rest of the community. Practically, local production would also reduce the cost of the cool roof because the transport and production costs were thought to be less in Burkina Faso.

*It has to be here*,* in Koussiri*,* in Kolonkoura or anywhere in Burkina*,* even if it is in Ouagadougou*,* it has to be based here. I don’t see the point of looking for it elsewhere. Not all people will be able to afford it. Some will also imitate others to get it because they will think that it is the heat pushed back by the roof of those who made the installation that they are cashing in. So*,* they will install it in their homes as well.* Woman, Trader.

*I would like it to be installed in Burkina Faso*,* it will be necessary to make an effort to have it installed here*,* otherwise when will you be able to find yourself in the West there. There*,* you and us here*,* we are moving from day to day between death and life*,* there will be a day when none of us lives any more*,* what will happen next? So*,* you’ll have to prepare the ground here in Africa for a job and for people to work there permanently and be qualified. That’s it*,* build a factory for that to facilitate production and acquisition. Otherwise*,* if it’s from the West*,* it’s complicated. We have to build a factory that makes them and hire our children.* Woman, Trader.

Local and community engagement were also considered the most useful and appropriate method to introduce people to the cool roof and make them aware of the product. Positive feedback from those with the roof would spread via word of mouth to family members, community leaders and local conversation within the community, promoting uptake and participation in the intervention.

*Information should circulate like this way of discussion that you are having with us*,* to present the benefits to them. We have to go through the village leaders*,* the village chiefs*,* the counsellors.* Woman, Farmer.

*We could pass information from family to family and from family to family*,* that’s it.* Man, Farmer.

*As soon as we see an example that is well done*,* people will disclose to each other that there is a system that is applied to the sheets to seal the heat. Then everyone will be able to buy it and use it. Because at home here*,* we learn about it by word of mouth.* Woman, Trader.

*My idea is a community thing*,* we want the whole town to benefit from it*,* we will pass on the information*,* you too can play a role*,* as the local radio is public*,* if we pass on a communiqué*,* there will be people who won’t have so much information*,* but if it’s through the radio*,* they will be informed and inform themselves. That too is a solution.* Man, Farmer.

### Opportunity costs

Opportunity costs are defined as ‘the extent to which benefits, profits, or values must be given up to engage in an intervention’ [23, 33]. The idea that benefits, profits, or values would be given up related to competing interests, limited availability of resources and to accepting something from the ‘white man’. As excessive heat was described as preventing participants from planting and harvesting as much as usual, and that this limited the ability to buy and sell goods, the benefits of the cool roof should be sustainable, in the sense of benefits being perceived to exceed that of any cost incurred by obtaining a cool roof. Participants also described scenarios where a sense of values relating to independence could be given up, because the intervention would be brought to the community and managed by the ‘white man’ who does not fully understand the suffering and reality of living with excessive heat. This would also make the community dependent on outsiders for maintenance of the roofs in the future, when greater value is given to self-reliance and community enpowerment.

*What I know is really salutary when it will be implemented but I draw your attention to the cost of the realization. In the previous agricultural season*,* the crops were not good and the sesame also did not give. There are also other expenses that we constantly have. So we’ll have to look at all of that. Since this is a start*,* if it starts out in good conditions*,* it will last*,* but if the conditions are not good*,* ah*,* there will be difficulties.* Man, Trader.

*Everyone does something out of interest*,* right? Well if they come to implement it and for ten years it doesn’t flow*,* after ten years it’s out of date and you can’t renew it and you can’t go back*,* it becomes a difficulty. That’s what we fear*,* we want peace.* Woman, Trader.

*You will obviously be relieved*,* it’s as if you leave under the burning sun to take refuge in the shade. That’s what we accept*,* but what we fear is that the white people*,* at some point*,* will trap us and create more difficulties for us.* Man, Farmer.

## Discussion

We present findings from 48 FGD participants to better understand the factors influencing the acceptability and preferences of a cool roof technology to be introduced in Nouna, Burkina Faso. To the best of our knowledge, this is the first study exploring the acceptability of cool roofs in Africa for adaptation of home occupants to increasing indoor heat exposure from climate change.

Our findings indicate that participants in Nouna, Burkina Faso will accept cool roofs provided they are effective at reducing indoor temperature and increasing occupant comfort. The greatest benefits of cool roof application were thought to apply to sheet metal roofs, which are becoming the common roof material used in rural Burkina Faso. Aesthetically, no changes were recommended prior to cool roof community implementation. Affordability and longevity of the cool roof were major considerations affecting acceptability. Local production and ownership of cool roofs were integral to build trust in the product, and promotion of the cool roof via word of mouth, discussions led by community elders and leaders were recommended for encouraging uptake.

We structured our findings around the acceptability framework of Sekhon, Cartwright and Francis (2017) [23, 33], to highlight the multiple components which influence the anticipated acceptability of the cool roof technology. Participants describe an environment of excessive heat and the need for adaptation strategies to achieve better wellbeing in Nouna. The cool roof would be acceptable if the cost, perceived effectiveness, values and ability to be self-efficacious within the trial– particularly in relation to local production and ownership– were suitable for the participants.

Our findings resonate with existing literature regarding climate adaptation interventions where acceptability is closely associated with cost and the perception of any burden the technology would place on those participating in the intervention [35, 36]. Low-cost interventions with proven benefits and minimal input requirements are popular, and ensure sustainability through community acceptability [37]. However, in other studies, costs were often related to the cost saving capacity of passive cooling technologies, as they reduce the need to use energy intensive cooling technologies such as fans or air conditioning [38]. As our participants live in one of the poorest countries globally and, of the 600 households enrolled in our prospective RCT, only 19 households have access to electricity and five have access to cooling fans, cost was only discussed in relation to affordability of the initial product and ability to maintain the cooling technology. In Burkina Faso, this is important because participants described the rising temperatures as a factor in reduced work productivity resulting in decreased income. The ability to earn money has been reduced because of the adverse impacts of heat exposure from climate change, yet the need for cooling is increasing. Therefore, willingness to pay for a heat reducing intervention could be a positive function of ambient temperature: the hotter the indoor temperature the more value the cooling technology has as cooler homes could increase productivity and potentially income. Willingness to pay may therefore increase due to the heat reducing function of the cool roof. Providing intervention participants with detailed information on upfront and maintenece costs will support a better understanding of whether the roof is accessible to home owners. We plan on conducting willingness-to-pay surveys alongside information regarding the– yet to be established - effectiveness of the cool roof, to help build a more holistic understanding.

As with work from Nielsen et al. (2010), we also see that tradition and values associated with housing and homes are closely linked with culture [39] and the environment [40], and that while new technology is acceptable, it is only required– not desired– due to the heat. The old, traditional banco houses are discussed with affection and belonging, and are not thought to be the reason for the increasing household temperatures or the associated health problems. This gives rise to a discussion about how the maintainence, promotion and continued construction of banco roofs could be supported to mitigate heat stress of people in rural Burkina Faso. Prior to application of cool roofs on banco roofs, we conducted adhesion testing and found that it was necessary to apply a primer to consolidate powdery mudbrick prior to cool roof coating application [41]. However, continued use of banco roofs maybe challenging considering houses can collapse from the build-up of weight during monsoon rainfalls. Banco roofs also require wood to provide structural support, whose availability is diminishing. Primer application worked well to enhance durability and minimise delamination of coatings on banco roofs.

As the inhabitants of Nouna are naturally migrating to metal roof use, one unintended consequence– and possibly an unintended burden - [42] is that the utilization of the cool roof technology may accelerate the uptake of the already broadly implemented sheet metal roofing. Those that have not yet switched to sheet metal roofs may do so, with the hope that they can have both the advantages of the metal roofs, without suffering the consequential heat, thanks to the cool roof technology. Thus, our study encourages sub-group-analysis of indoor temperatures in banco-roof versus tin-roofs with and without the cool roof technology to provide information in how far the roofing type influences indoor temperatures.

The fear that the cool roof could trap the participants in a long-term relationship with the ‘white man’ who had created a product without due consideration for the people that would use the product was a clear and consistent theme. We linked this to the construct of self-efficacy because this highlights where participants have reservations regarding the intervention, because it may not be designed for them or their context. This resonates with work from Yamey (2012) [43], where local ownership and production were important for acceptability because of the independence from external parties. This independence - in a community well versed in building and maintaining roofs - plus the additional boost for the economy, and the new information and learning that could be gained from the cool roof, could be a point to leverage cool roof acceptability. However, as other widely accepted products such as metal roofs and plastic buckets are also not produced locally, ‘ownership’ may be more about availability, and having the capacity and independence to maintain the roofs, rather than being directly related to production.

The theme of independence is evident in intervention research, where the sustainability of interventions are seen as more likely when local actors [44], structures [45] and governance [45] are integrated early in the project. Building capacity within the community via sharing knowledge and ownership was welcomed by our participants, and is shown elsewhere to ensure the maintenance, development, expansion and improvement of interventions over the long term [44]. This idea extended to how the community could be introduced to the cool roof. Our participants preferred to be introduced to the technology through word of mouth via family, community leaders and community channels, and felt that radio could play a complimentary role. Community engagement using respected community leaders has been considered the most important method of promoting a product to– especially rural– communities [46], and although underused [47], should be an essential component of the introduction of health-related interventions [48].

Preferences regarding the cool roof were aligned with functionality: white cool roofs have the highest reflectivity, offering the greatest cooling potential, and as the majority of our participants accepted white as a suitable colour, this– as long as it is visible - will stay as the colour used in future applications. An important co-benefit would be if the cool roof had waterproofing and noise reduction properties. Providing co-benefit information in addition to first-hand experience of the integrity and function of the materials - including the durability, longevity and functionality– may be appropriate approaches to achieving acceptability of the roof. This information should be clear for cool roof users, alongside a systematic dissemination of information regarding the cooling effects of the roof with the participants and wider community.

While the Sekhon, Cartwright and Francis (2017) [23, 33] framework has been a useful tool in building our understanding of acceptability, our current study does not report on cool roof desirability. As detailed by Kim (2013) [49], desirability should be a fundamental aim of the promotion of any product [50], because acceptability is a passive minimum standard to achieve in Burkina Faso, whereas desirability means people are actively seeking the product [51]. Following the use of cool roofs and the conclusion of the RCT, we will report on desirability of cool roofs, following what our framework highlights as the key areas that appear to feed into the idea of desirability, especially where self-efficacy, intervention coherence and perceived effectiveness are concerned. These three areas show the importance of marketing a product alongside the values associated with belonging, ownership and independence, as well as the cooling data as it becomes available– in the form of personal accounts as well as temperature measurement data– and details on the quality and functionality of the product. Integrating these elements as a marketing tool within the intervention now, and building them from the beginning of projects– in Burkina and globally– may ensure the rapid and desired uptake of a passive cooling technology designed to improve the health and life quality of some of the worlds poorest individuals.

### Limitations

Our study was conducted with participants that had not yet received the cool roof intervention, so our framework can only capture the anticipated cognitive and emotional responses to the intervention. In order to better understand acceptability, preferences and desirability, we will conduct further FGDs, in-depth interviews and hold participatory design meetings once the intervention has been received, with homeowners, sharing cooling data and experiences as they emerge. We initially selected participants who indicated they had either banco or sheet metal roofs, aiming for equal representation. However, during the focus group discussions, some participants who identified as having banco roofs also mentioned having sheet metal. This discrepancy arose because “home” often referred to multiple buildings, with participants’ point of reference shifting based on the activity (e.g., cooking versus sleeping). Upon analyzing the transcripts, it became clear that most participants had both types of roofs, with only two participants having homes exclusively with banco roofing.

## Conclusions

This qualitative study was conducted as part of an ongoing RCT in Nouna, Burkina Faso. The FGDs gathered insights regarding perceptions and the acceptability of the cool roof paint prior to the intervention. We found that the acceptability of cool roofs was closely linked with the perceived ease of participating in the intervention, the perceived effectiveness of the cooling technology, affordability and its alignment with values, history, belonging and self-efficacy. Acceptability and uptake of cool roofs in Nouna will depend on providing communities with technical information regarding the product, including how it functions and maintenance requirements. No changes to the product (i.e., colour) were deemed necessary. Making the roof desirable may entail a combination of highlighting the co-benefits of the roof alongside the cooling as reported by those using it, sharing data with the community so that they have an insight into the cooling effects as recorded by the study instruments, and feedback regarding the products useability and durability.

## Figures and Tables

**Figure:**
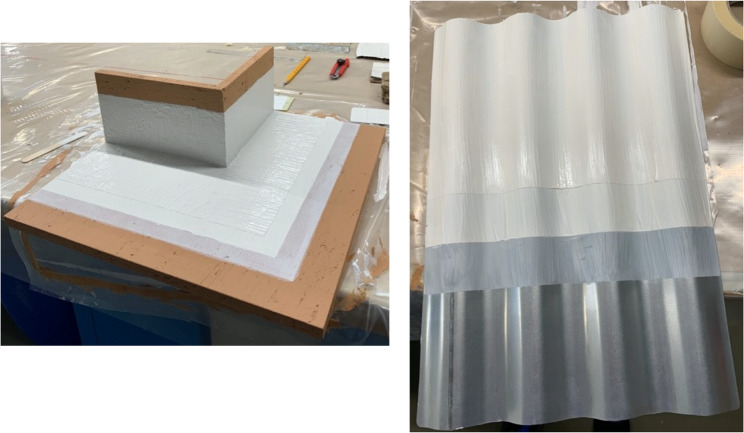
**a** mudbrick roof with cool roof layers visible. **b** metal roof with cool roof layers visible

## Data Availability

Data can be made available upon request from A.B. Please request the data using this email address: aditi.bunker@uni-heidelberg.de.

## Appendix

### Focus Group Discussion Guide (integrating elements relevant to fulfill data needs for study 1)

As we have discussed earlier, prior to signing the informed consent, we are here to talk to you about your feelings and preferences towards weather, weather changes and variability as well as cool roof technology. I will put out a few points for discussion, but please feel free to contribute any additional thoughts you may have on the matter. We are here to learn from you and your experience.

What is climate and how do you think about climate?

What is your understanding of climate change? Can you provide any personal examples of climate change you have experienced? Do you think there is a link between climate change and health– please elaborate: why or why not? What does climate change mean for you and your community?

1. Let us start by looking at your level of heat stress at home and during work. Can you tell me how you experience heat stress? Do you feel that heat stress limits your working abilities?
2. Can you explain what you do to prevent yourself from heat-stress at home and during work? Are there any events external to your control that may make you specifically stressed by higher temperatures? If so, which ones are these elements? How often do they occur?
3. Does your household or your community more in general have any means of protecting oneself against heat-stress? If yes, which ones? (focus really on the capacity to prevent the adverse event not to cope with its resulting losses).
4. What happens to your household when heat-stress becomes too strong to continue working or staying at home? How do you cope with these losses in resting time or working hours? What strategies do you implement at household or community level? How effective are these strategies?
5. What do you think of your current roof material? What type of roof material would you prefer to have? Explain your current practices for maintaining your roof, i.e., do you apply any substances regularly to your roof? We know there is the practice of applying the *burre de karité *once a year to waterproof your roof for the rainy season - what is the cultural significance of this practice to you? Would it be good or bad if you didn’t have to do it anymore?

Some people in Burkina Faso (i.e., the government and its development partners) are talking about introducing those cool roofs to offer people the possibility to limit the adverse effect of heat on losses on the household wellbeing due to extreme changes in weather. The basic idea of these roofs is to put a thin coating like a paint on the roofs to increase the reflectance of the sun rays. This can help to cool down the indoor temperature and thereby, reduce heat-stress to the people who live there.

Here we have a demonstration prop showing two types of roofs: a) mudbrick and b) metal roofs. Please have a close look– touch it and feel it. First the roof will be cleaned to remove any particles on the roof. Multiple layers of coatings will be applied to create a cool roof. The first layer is called the primer - the paint is diluted with a little water to create strong adhesion and seal the pores of the mudbrick or metal roof. Then two more layers of undiluted paint are applied to create a final cool roof, which has high sunlight reflection and waterproofing properties. This means it is unlikely water will leak into your home. The product will stay on for a long time– at least 3 years to 10 years depending on environmental factors like wind, rain, dust etc. The products are water based and safe for human contact.

We are curious to hear your opinion on the possibility that such a roof would be offered to your household.

6. Have you ever heard of such a cool roof from anyone? If so, from whom and under what circumstances? Do you know any other kinds of these roofs? Were people in your community offered such a cool roof technology? With what experience? What do you know about cool-roof technologies?
7. What are your immediate thoughts on such a cool roof linking weather variability with health? In which ways, if any, do you think that such a cool roof would be useful in your home?
8. Do you think that the installation of such a cool roof could works in your home? Why? Do you think that local communities would be likely to get these installations themselves? Do you think that people would be likely to pay a bit of money to receive these cool roofs? Why? Do you think that people in your community would trust such a product if promoted by national government?
9. If such a roof should come to your house, it would be important that its features are well aligned with the needs and preferences of the people who live here. Once the roof paint is applied, we will measure different outcomes to see if a cool roof is useful for people’s health and to make the environment in the home more comfortable. So, one by one, we would like to ask you to help us think of how people will react to features of the cool roof and how we can take health and environmental measurements that people will accept
  - Material and color of the roof
  - Measurements to assess indoor and outdoor temperatures and humidity
  - Measurements to assess health status
  - Possibility to pay for the cool roof
10. If such a cool roof were to come to your home, how do you think that this should be done? What messages do you believe should be used to advocate this to the whole community? And whom do you think should conduct the promotion campaign?
11. Here is a before and after image of the what a village could look like with cool roofs. What are your thoughts on this?

12. Are there any other points you think are relevant for discussion and that we have not yet raised?
